# Opportunistic Autoimmune Disorders Potentiated by Immune-Checkpoint Inhibitors Anti-CTLA-4 and Anti-PD-1

**DOI:** 10.3389/fimmu.2014.00206

**Published:** 2014-05-16

**Authors:** Yi-chi M. Kong, Jeffrey C. Flynn

**Affiliations:** ^1^Department of Immunology and Microbiology, Wayne State University School of Medicine, Detroit, MI, USA; ^2^Department of Orthopaedic Surgery, Providence Hospital and Medical Centers, Southfield, MI, USA

**Keywords:** anti-CTLA-4, anti-PD-1, autoimmune disease, tumor immunity, immune-checkpoint inhibitor

## Abstract

To improve the efficacy of immunotherapy for cancer and autoimmune diseases, recent ongoing and completed clinical trials have focused on specific targets to redirect the immune network toward eradicating a variety of tumors and ameliorating the self-destructive process. In a previous review, both systemic immunomodulators and monoclonal antibodies (mAbs), anti-CTLA-4, and anti-CD52, were discussed regarding therapeutics and autoimmune sequelae, as well as predisposing factors known to exacerbate immune-related adverse events (irAEs). This review will focus on immune-checkpoint inhibitors, and the data from most clinical trials involve blockade with anti-CTLA-4 such as ipilimumab. However, despite the mild to severe irAEs observed with ipilimumab in ~60% of patients, overall survival (OS) averaged ~22–25% at 3–5 years. To boost OS, other mAbs targeting programed death-1 and its ligand are undergoing clinical trials as monotherapy or dual therapy with anti-CTLA-4. Therapeutic combinations may generate different spectrum of opportunistic autoimmune disorders. To simulate clinical scenarios, we have applied regulatory T cell perturbation to murine models combined to examine the balance between thyroid autoimmunity and tumor-specific immunity.

## Introduction

In recent decades, cancer therapy has focused on amplifying the immune system to bolster the host’s anti-tumor response. Utilizing systemic immunomodulators, monoclonal antibodies (mAbs), and stem cell transplantation, progress has been rapid in prolonging survival. However, targeted immunotherapy has come with a price; altered immunoregulation provoking immune dysfunction has opened the door to opportunistic autoimmune disorders. Previously, both systemic immunomodulators and mAbs, anti-CTLA-4 and anti-CD52, were discussed in terms of therapeutic usage, autoimmune sequelae, and important predisposing factors, e.g., *HLA* class II genes and gender predilection, known to influence immune-related adverse events (irAEs) (1). Similar and additional immunotherapeutic modalities associated with autoimmunity, particularly thyroid dysfunction, were likewise highlighted by others (2, 3). In addition to CTLA-4, a number of immune-checkpoints are also being targeted in cancer immunotherapy. Thus far, the most information has come from longer and larger clinical trials with anti-CTLA-4 (ipilimumab and tremelimumab), accompanied by mild to severe irAEs (4–6). In early trials, it was hoped that irAEs could serve as a predictor of improving anti-CTLA-4 immunotherapy (7, 8). However, analysis of 139 metastatic melanoma patients given ipilimumab revealed that the frequency of irAEs after a 2–4 year follow-up was 81% with a total response rate of 17% (23 of 139) (7); of the 86 patients with irAEs, 74% (64 of 86) showed no objective improvement (1).

Thus, targeting CTLA-4, a T cell regulatory molecule, impacts on its two primary functions in the immune network: (1) Its upregulation during a T cell-mediated response serves as a negative regulator by engaging the B7 family costimulatory molecules on antigen-presenting cells with higher avidity than CD28 (9); and (2) Its constitutive expression on regulatory T cells (Tregs) is critical to the Foxp3 function in suppressing autoreactive T cell activation (10, 11). *ctla4^−/−^* mice develop severe multiorgan autoimmunity, indicative of deficiency in both these functions (12, 13). When humanized CTLA-4 mAbs were first used to treat advanced melanoma a decade ago, the major goal was to interfere with the negative signaling of an ongoing anti-tumor response discernible in many patients (4, 6). However, since a key role of CTLA-4 is to enable Treg suppression of autoreactive T cell activation at the costimulatory level of DC (10, 11), it was no surprise that opportunistic autoimmune disorders surfaced as prominent irAEs from CTLA-4 blockade. The variety stems from the ever-present autoantigens and autoreactive T cells unleashed from self tolerance regulation (1). For example, in our tolerance induction study in murine experimental autoimmune thyroiditis (EAT), a model for the prevalent Hashimoto’s thyroiditis (14), co-injection of anti-CTLA-4 with the autoantigen, thyroglobulin (Tg), interfered with activation of naturally existing CD4^+^CD25^+^Foxp3^+^ Tregs (nTregs); the mice developed thyroiditis, mimicking a major clinical autoimmune sequela (15).

Using revised assessment criteria including overall survival (OS) in phase II/III trials to take into account the longer survival kinetics for ipilimumab (16), recent compilation of ipilimumab phase I/II trial results showed a range of 12–36% OS at 3–5 years, with variables including the dose, patient number, prior, or adjunct treatment (4). Pooling phase II/III trials showed irAEs approximating 60% with less severe grade 3–4 in the phase III trials, likely due to earlier recognition and management of autoimmune sequelae (4, 17). While the percentages of irAEs varied, most included skin rashes, colitis, thyroid dysfunction, hypophysitis, hepatitis, and pancreatitis (4, 17), as also reported in western Europe (5). Treatment-related deaths continued to occur and severe morbidity required stringent life-long treatment and hormonal supplementation (4, 5). The second mAb, tremelimumab, likewise underwent phase I/II trials [see Ref. (1)] and phase III trials with similar irAEs but less durable OS than with ipilimumab; in fact, survival was not much longer than after standard chemotherapy with temozolomide or dacarbazine (6). To boost OS, mAbs that blockade the function of another immune-checkpoint, programed death-1 (PD-1), or its ligand (PD-L1), have been undergoing clinical trials as monotherapy or dual therapy with anti-CTLA-4.

## Models to Probe the Balance between Autoimmunity and Tumor Immunity upon Treg Perturbation

The high percentages of irAEs from anti-CTLA-4 therapy clearly show that maintenance of Treg function and self tolerance constitutes a premier CTLA-4 function. Autoimmune thyroid disease, including Hashimoto’s thyroiditis and Graves’ disease, represents the most prevalent autoimmune condition (18), and CTLA-4 blockade has joined other systemic immunomodulators [e.g., interferon-α, -β, interleukin (IL)-2] and leukocyte-target agents (e.g., anti-CD52) in triggering thyroid dysfunction (1, 3, 19, 20). As EAT has long served as a model to study Treg function in self tolerance to mouse Tg (14, 21, 22), we developed in recent years four murine models combining EAT and breast cancer vaccine protocols under Treg perturbation and MHC class II gene influence, using autoimmune thyroiditis as a sequela indicator (23).

The first three utilized well-established tumor models in wild type mice or mice transgenic for Her-2/neu breast cancer antigen, which harbor class II-linked, EAT-resistant haplotype (*H2^d^* or *H2^b^*). In the first model, we induced EAT concurrently with anti-tumor immunity in wild type mice at the time of Treg depletion with mAb to CD25 (24). Treg depletion enhanced tumor regression and thyroiditis. Immune responses to neu and mouse Tg were greater than control groups given tumor or Tg alone, indicating that ongoing tumor regression and autoimmune response provided additional mutual stimuli. In the second model, we used rat neu-transgenic mice, which required both Treg depletion and neu DNA vaccination to develop resistance to tumor challenge and spontaneous tumorigenesis (25). Mutual stimulation during responses to neu and mouse Tg was again observed. In tumor-regressing mice, there were significant increases in interferon-γ-producing T cells and greater thyroid destruction even in the EAT-resistant strain. Lastly, in the third model, we introduced the HLA-DR3 transgene, an EAT-susceptible allele (26), and an Her-2 transgene into EAT-resistant *H2A^b^* mice to determine if anti-tumor response was independent of EAT susceptibility. (*A^b^*-Her-2xDR3)F_1_ mice expressed both A^b^ and DR3 and were tolerant to both Tg and Her-2 (27). After Treg depletion followed by Her-2 DNA and mouse Tg injections, tumor rejection was similar in Her-2 transgenic mice expressing either A^b^ or A^b^/DR3, but thyroiditis was augmented only in (*A^b^*/DR3)F_1_ mice, showing that Her-2 immunity, unlike autoimmunity, was independent of DR3 expression.

In the fourth combined model, we used an EAT-susceptible CBA/J (*H2^k^*) haplotype with demonstrated antigen-specific nTreg-mediated tolerance. Depleting pre-existing nTregs markedly enhanced thyroiditis development with soluble mouse Tg even without adjuvant (15). Control mice with nTregs, which had been activated/expanded after exposure to mouse Tg by either injection or physiologic release (via thyroid-stimulating hormone infusion in an osmotic pump), withstood EAT induction with mouse Tg plus adjuvant (22, 28). In contrast, nTreg-depleted mice were incapable of establishing this strong and long-lasting tolerance (14, 15, 22). For the cancer portion, CBA/J tumor was derived from a spontaneous mammary adenocarcinoma line, and resistance to lethal challenge was instilled by prior Treg depletion and vaccination with irradiated tumor cells (23). To simulate patients with MHC class II-associated predisposition to autoimmunity and subjected to immune targeting, mouse Tg was also given. Treg depletion not only augmented tumor immunity but also thyroidal infiltration (29). Furthermore, to simulate the scenario in some cancer patients with pre-existing autoimmunity and given immunotherapy, mice were pretreated with mouse Tg + low doses of IL-1 to establish a subclinical, mild thyroiditis condition. Treg depletion, tumor vaccination, and mouse Tg injections then followed. While anti-tumor immunity remained unchanged, thyroiditis was exacerbated (29). Thus, this recent model takes into account genetically predisposed patients who have no underlying thyroid dysfunction or have pre-existing, undiagnosed disease.

## Targeting CTLA-4 led to Unusual Spectrum of Autoimmune Sequelae

In murine EAT, tolerance induction with the known autoantigen, mouse Tg, and its blockade of nTreg activation by anti-CTLA-4 to allow thyroiditis development can be followed with timed co-administration (15). However, in cancer patients, there are multiple self antigens for which the maintenance mechanisms of self tolerance can be disrupted with anti-CTLA-4 therapy at varying doses and intervals, resulting in unpredictable manifestations of 20–60% of irAEs with grade 1–4 severity. In addition to advanced melanoma, both ipilimumab and tremelimumab have been used to treat other solid tumors and hematologic cancers and the frequent irAEs have included dermatitis, enterocolitis, hepatitis, thyroid dysfunction, and hypophysitis with widespread endocrinopathies, irrespective of cancer types (3–6, 19, 20). Although these autoimmune diseases are primarily organ-specific, T cell-mediated, and with mononuclear cell infiltration of the specific organ, plasma cells may be seen with time. Moreover, when several organs are involved with attendant proinflammatory cytokines, irAEs may become exacerbated and more widespread. Thus, different irAEs often occur throughout the treatment period and beyond.

There is agreement that the extent of severe irAEs is dose-dependent, but OS improvement generally does not correspond linearly with dosage (5), nor correlates with severity of irAEs (1, 19). Torino et al. (19) undertook an in-depth analysis of endocrine dysfunction for 21 phase I–III trials with ipilimumab treatment of melanoma and several other solid tumors. The primary emphasis was on hypophysitis of grades 3–4, but the involvement of other endocrine dysfunctions (hypothyroidism, adrenal insufficiency) and other common irAEs (e.g., diarrhea, colitis/enterocolitis, dermatitis, hepatitis, and arthritis) was also included. A more recent report by some of the same authors, Corsello et al. (20), extended the analysis to 28 trials, subcategorizing classifiable endocrine disorders and other common irAEs, which may or may not be autoimmune in origin. As mentioned above, since irAEs generally involved up to 60% of patients, at 2–3× the OS, a direct correlation of OS with irAE would be difficult at this time (4).

As discussed earlier (1), while the increase in thyroid dysfunction could have been anticipated, given the high prevalence in the general population, the most unusual and distinct association with anti-CTLA-4 therapy is autoimmune hypophysitis, with an incidence of 3–9% in early trials far exceeding its rare occurrence (~1 in 0.5 million) (19, 30). Another early melanoma trial with ipilimumab reported 17% (8 of 46) (31). The analyses of clinical trials with ipilimumab and tremelimumab gave an average of ~4% hypophysitis, closely rivaling hypothyroidism (19, 20). While hypothyroidism is 5–6× more prevalent in females in Caucasians (18), thus far hypophysitis from anti-CTLA-4 therapy has occurred mostly in males, in part influenced by the ratio of female:male of ~1:2 in melanoma patients. Other genetic (HLA, CTLA-4 gene polymorphism, mutations) and environmental factors may also take part (1, 4, 32). The high incidence of hypophysitis, its need for early corticosteroid intervention and life-long hormonal replacement prompted the advocacy of early recognition and management, together with thyroid function tests (17, 19, 20). Searches for specific pituitary antigens for early antibody detection to replace immunofluorescence on pituitary sections are ongoing (33–35).

Although autoimmune sequelae (e.g., hypophysitis, thyroid dysfunction, and hormonal imbalance) are gaining recognition, the high incidences of colitis and dermatitis have not been investigated as to culprit antigens, which could be self, commensally microbial, or tumor-associated. A recent survey of anti-CTLA-4 therapy in 752 melanoma patients at 19 skin centers in western Europe excluded colitis and dermatitis from the usually observed 60% irAEs (4, 5), and concentrated on raising awareness to rare yet severe toxicities, some of which may be related to prior chemo- or radio-therapy. A total of 88 irAEs from 82 of 752 (11%) patients were tabulated, 12 with hypophysitis (5). With melanoma patients, the side effects of vitiligo and uveitis were often noted because of shared melanocyte antigens, often aggravated by vaccination (1, 5). The 15 rarer cases included gastric problems, ischemia and bowel perforations, fatal hepatitis, hypophysitis with brain edema, and inflammation of the central nervous system. There were also respiratory and cardiac problems. Thus, the untoward autoimmune disorders are opportunistic and unpredictable, as well as far-reaching.

## Specific Antigens to Boost Tumor Immunity and Monitor Treg Activity

As seen above, the weakly immunogenic, tumor-associated antigens, which can be overexpressed self, altered self, or neoantigens, are further interwoven with unexpected pathogenic autoantigens during CTLA-4 blockade. Examination of tumor infiltrates after tremelimumab therapy revealed an increase in activated CD8^+^ T cells in many melanoma patients; while not Tregs, this non-specific increase was observed in a greater proportion of patients than patients deriving any clinical benefit and showed no correlation with either tumor progression or regression (36). In phase II trials of melanoma patients with ipilimumab, a peripheral increase in the %CD4^+^ and %CD8^+^ activated T cells, but not those with a Treg phenotype, was noted (37). Of interest was the greater serologic reactivity to several melanoma antigens and a cancer-testis antigen NY-ESO-1. Because NY-ESO-1 is expressed in a number of solid tumors and 30–40% of advanced melanoma patients, it has been examined as a specific biomarker for increased T and B cell reactivity after CTLA-4 blockade, and if such reactivities could be associated with positive clinical outcome (37–40). While an increase in anti-NY-ESO-1 has often been detected, its association with positive benefit is still controversial (38–40). However, it appeared that clinical benefit could be associated with NY-ESO-1 reactivity if both antibody and CD8^+^ T cells were considered in concert (10 of 13 or 77%) (39). More such studies will be needed to determine if specific reactivity to NY-ESO-1 as well as other melanoma peptides has a predictive value. Meanwhile, to increase the efficacy of CTLA-4 blockade and detect changes in tumor-specific effector T cells, therapeutic vaccines are being incorporated. In a large phase III trial (41), a gp100 peptide vaccine was given with ipilimumab (403 patients), compared to ipilimumab (137) or gp100 (136) alone. However, there was no significant increase in OS provided by including gp100 vaccination, compared to ipilimumab alone.

Detecting antigen-specific effector T cells is complicated by the presence of Tregs. In the tumor microenvironment, induced Tregs (iTregs) represent a major component of suppressor cell subsets and, as opposed to nTregs, usually arise or are converted from naive T cells in response to antigenic stimulus plus local TGF-β and IL-10 production (42). Intratumoral iTregs often exist in greater percentages than in the periphery. Although in murine melanoma studies Fc-dependent ADCC-like depletion of Tregs was demonstrable upon anti-CTLA treatment (43), the frequent detection of intratumoral iTregs in the human suggests that this kind of depletion might be an adjunct to improved clinical benefit. But the challenge remains to identify whether the effect was on tumor-specific iTregs, so as to monitor and inhibit their suppressor role (44). Following therapeutic vaccination, multiple peptides have been used to monitor iTreg activity. In one phase II trial in renal cell cancer patients, cyclophosphamide, known to deplete Tregs (22), was given before the IMA901 vaccine, consisting of multiple tumor-associated peptides used to demonstrate heightened T cell responses (45). In another study, synthetic peptides were used to compare the repertoire of tumor-specific iTregs and effector T cells in colorectal carcinoma patients (46). While differences were noted between the two populations, the suppressor activity was shown to stem from iTregs that shared the same repertoire as effector T cells. Additional studies have made use of class II tetramers to monitor antigen specificity. After vaccination of melanoma patients with HLA-A2-restricted Melan-A peptide, monitoring with HLA-DQ6-restricted Melan-A peptide multimers revealed a downshift in specific Tregs with increased effector T cell responses (47). In breast cancer patients, tetramers from HLA-DR4-, DR7-restricted mammaglobin peptides were used to detect iTregs in the periphery (48). After their depletion *in vitro*, effector T cell responses to several mammaglobin peptides increased. Thus, antigenic specificity, if known, could aid assessment of immunotherapeutic efficacy.

## PD-1 and PD-L1 Blockade to Augment Tumor Immunity

Recent reviews have discussed in depth the phenomenon of T cell exhaustion, where activated T cells highly expressing PD-1, as exemplified by virus-specific CD8^+^ cells, exhibited exhaustion phenotypes and failed to combat chronic infections (49–51). In the tumor microenvironment, PD-1 expression was found on impaired infiltrating lymphocytes (52) and Tregs (53). Its major ligand, PD-L1, has been found on multiple epithelial carcinomas, compared to PD-L2 on lymphoid tumors associated with its expression pattern (49–51). There is no clear consensus on whether PD-L1 expression by tumors is associated with greater objective response from PD-1 blockade; more specific staining reagents have led to reports of positive association (54, 55). PD-1 is also expressed on activated B, NK, and NKT cells and is involved in down-regulating autoimmunity. PD-1-deficient C57BL/6 mice exhibited lupus-like arthritis and glomerulonephritis (56), and PD-1-deficient BALB/c mice developed cardiomyopathy from an autoantibody to cardiac troponin 1 (57). Thus, these autoimmune syndromes have a strong pathogenic autoantibody component. While preclinical tumor models have shown clinical benefit with PD-1 blockade, it is uncertain if only activated CD8^+^ cells participated in the OS (50).

Two phase I, ongoing dose-escalation trials have been reported with two IgG4 mAbs to PD-1. Lambrolizumab (MK-3475), which has recently been designated by the FDA as a “breakthrough therapy” drug to treat advanced melanoma (58), was used to treat 135 melanoma patients, who were evaluated for up to 70 weeks (59). The confirmed response rate was 38% (44 of 117), irrespective of prior ipilimumab treatment, and appeared dose-dependent. Biopsies of regressing lesions revealed densely infiltrated CD8^+^ T cells. irAEs were 79% (107 of 135); 17 of the 107 had grade 3–4. Beside skin and gastrointestinal problems, hypothyroidism was 8%. The second phase I trial with nivolumab (BMS-936558) included 296 patients with melanoma, non-small cell lung cancer, prostate cancer, renal cell, or colorectal cancer. The first report in 2012 was after ~1 year and the objective response averaged ~20–25% (55). Grade 3–4 irAEs were observed in 14% (41 of 296), of which 11% were considered serious; 15 patients discontinued the study and 3 deaths (1%) were attributed to pneumonitis. The spectrum of irAEs was mostly similar to lambrolizumab therapy (59). But with twice the number of patients, the variety of irAEs resembled those seen with ipilimumab, albeit at lower overall percentages. The spectrum was dose-dependent and included rash, pruritus, and diarrhea (all at ~27%), with hyperthyroidism and hypothyroidism at 3–7% (55). An exception to the ipilimumab treatment was the prominent pulmonary toxicities seen in 2–4% of patients (55, 59). In addition to fatalities (55), the 2–4% in phase I trials were of grade 3–4 (55, 59). A recent report about ongoing phase II/III trials also listed pneumonitis as a notable side effect (60). Whether it is autoimmune in origin is undetermined. An updated analysis on 107 melanoma patients treated with nivolumab showed 40% OS at 3 years (61).

Another multicenter phase I trial enrolled 207 patients without prior experience with CTLA-4, PD-1, or PD-L1 mAb, who were given anti-PD-L1 (IgG4, BMS-936559) for a number of solid tumors for up to 2 years (62). The durable response rate was 6–17%. Treatment-related adverse events were noted in 61% (126 of 207), and 39% (81 of 207) were considered irAEs; these patients have a somewhat different spectrum and a lower percentage of irAEs compared to patients in the anti-PD-1 trials. Because these investigators were also conducting the phase I trial with nivolumab (55), their initial clinical impression was that anti-PD-L1 blockade was inferior to anti-PD-1 in achieving objective responses (62).

Interestingly, pneumonitis was not a noted side effect in the anti-PD-L1 phase I trials (60, 62), but the 39% irAEs showed a distinct autoimmune-related trend: rash, hypothyroidism, hepatitis, plus isolated cases of diabetes mellitus, and myasthenia gravis, all mostly of grade 1–2 (62). PD-1 binds to both PD-L1 (broad tissue distribution) and PD-L2 (limited primarily to DCs) (63, 64). Since lung tissues express PD-L1 and contain activated alveolar macrophages, it is possible that anti-PD-1 blockade removes the inhibitory signals that control tissue proliferation and cytokine production more so in the lung, resulting in pneumonitis, whereas anti-PD-L1 blockade does not block the immune-checkpoint between PD-1 and PD-L2. Another possibility is that, since self tolerance is maintained by the continuing interaction between PD-1 and PD-L1 to prevent TCR-driven signaling (65), upon anti-PD-L1 blockade, autoreactive T cells could become activated resulting in the autoimmune syndromes reported.

## Combining Immune-Checkpoint Inhibitors Could Potentiate Autoimmune Sequelae

There are multiple national clinical trials planned or ongoing with ipilimumab plus chemo- or radio-therapeutic agents, cytokines (e.g., GM-CSF, IL-2, IL-21), and other systemic immunomodulators, most with the goal of stimulating the effector T cell arm with some targeting dendritic and B cells also [see Ref. (51)]. Because anti-CTLA inhibited Treg function and potentiated irAEs, the use of low dose cyclophosphamide could further target nTregs (22, 45) and increase autoimmune sequelae, similar to Treg-depleting anti-CD52 (1). In murine models of B16 melanoma tumors (66) and CT26 colon and ID8-VEGF ovarian carcinomas (67), dual blockade of CTLA-4 and PD-1 enhanced greater tumor rejection than each alone. It should be noted that, unlike in clinical trials, these tumors were manipulated to express GM-CSF, as was whole cell vaccination included in some experiments. The presence of GM-CSF could influence the expression pattern reported for tumor-infiltrating lymphocytes (67). Table 1 lists the important functions of CTLA-4 and PD-1 molecules in maintaining homeostasis of the immune network, and provides examples of the impact of anti-CTLA-4 and/or anti-PD-1 blockade on tumor immunity and autoimmunity.

**Table 1:** **Function of CTLA-4 and PD-1 in the immune network and the impact of immune-checkpoint inhibitors anti-CTLA-4 and/or anti-PD-1 on examples of tumor immunity and autoimmunity**.

Functions	Consequences	Examples of anti-CTLA-4 and/or anti-PD-1 blockade on	
	
		Tumor immunity	Autoimmunity
CTLA-4 upregulation on APCs/peptide-stimulated Tregs downmodulates B7-1/B7-2 on APCs	Suppresses priming of naive/autoreactive T cells and maintains peripheral tolerance		Morris et al. (15): EAT
			Read et al. (70): colitis
			Ansari et al. (72): type I diabetes
CTLA-4 binding to B7-1 and B7-2 causes reverse signaling through B7-1/B7-2	Activation of the tryptophan-catabolizing enzyme indoleamine 2,3-dioxygenase inhibits T cell priming and proliferation	Holmgaard et al. (68): melanoma	Kwidzinski et al. (73): EAE
CTLA-4 signaling stimulates production of cytokines TGF-β and/or IL-10 by Tregs	Inhibits function of APCs and T cells		Liu et al. (75): colitis
CTLA-4 upregulation on activated T cells binds to B7-1/B7-2 at high affinity	Negative feedback signaling inhibits continued T cell proliferation	Leach et al. (69): colon carcinoma	Oaks and Hallett (76): AITD
		Hurwitz et al. (71): prostate cancer	Hurwitz et al. (71): prostatitis
			Choi et al. (77): CIA
			Hurwitz et al. (78): EAE
			Torino et al. (19): clinical hypophysitis
	Fc-dependent depletion of iTregs	Simpson et al. (43) melanoma	
CTLA-4 signaling alters motility and inhibits T cell receptor-mediated “stop” signal	Reduces efficiency of effector T cell killing and APC interaction	Ruocco et al. (74): breast cancer	
PD-1 signaling enhances Treg function	Inhibits T cell priming and maintains self tolerance		Ansari et al. (72): type I diabetes
PD-1 signaling inhibits motility and T cell receptor-mediated “stop” signal	Inhibits autoreactive T cell activation and reduces effector T cell function	Holmgaard et al. (68): melanoma	Fife et al. (65): type I diabetes
PD-1 upregulation on activated T cells	Inhibits effector T cell function (anergy or exhaustion)	Holmgaard et al. (68): melanoma	Ansari et al. (72) type I diabetes
		Ahmadzadeh et al. (52): melanoma patients	
		Wang et al. (53): melanoma patients	
Dual blockade		Duraiswamy et al. (67): colon and ovarian cancers	
		Curran et al. (66): melanoma	

*AITD, human autoimmune thyroid disease; APC, antigen-presenting cell; CIA, collagen-induced arthritis; EAE, experimental autoimmune encephalomyelitis; EAT, experimental autoimmune thyroiditis; iTreg, induced regulatory T cell; Treg, regulatory T cell*.

Since CTLA-4 blockade interferes with peripheral tolerance induction/maintenance and affects primarily the early stages of the immune response, and PD-1 blockade acts toward the late stages at the tissue sites (79) and each can augment OS, dual therapy could further enhance OS, provided that the irAEs are not unreasonably additive. In melanoma patients, a phase I trial combining ipilimumab and nivolumab has begun (80). The need to test different regimens resulted in small patient sizes of 33–53. While the spectrum of irAEs was essentially similar to monotherapy, OS was higher at 24 weeks, based on previous experience.

Longer follow-ups and additional trials will be necessary to assess various parameters affecting irAEs: HLA genotype, environmental and gender influences, and antigen specificities for both Tregs and effector T cells. As seen in our EAT-anti-tumor models, subclinical autoimmune conditions (29), and mutual stimulation arising from anti-tumor and autoimmune inflammation also contribute to the overall response enhancing tumor immunity and autoimmunity (24, 25). Importantly, there appears to be different pathogenic pathways to autoimmune manifestations, with PD-1-deficiency favoring a pathogenic autoantibody profile and CTLA-4 blockade favoring T cell-mediated organ damage.

## Conflict of Interest Statement

The authors declare that the research was conducted in the absence of any commercial or financial relationships that could be construed as a potential conflict of interest.
